# Celecoxib suppresses colorectal cancer growth by inhibiting propionyl–CoA carboxylase alpha chain-mediated epithelial–mesenchymal transition and angiogenesis

**DOI:** 10.1093/gastro/goag071

**Published:** 2026-08-10

**Authors:** Chuyi Zhang, Jielin Zheng, Yingyin Zhao, Fai Leong, Jiahe Xu, Zhunyi Gao, Ziwei Qi, Yuhui Huang, Sanqing Jin

**Affiliations:** Department of Anaesthesia, The Sixth Affiliated Hospital, Sun Yat-sen University, Guangzhou, Guangdong 510655, P. R. China; Biomedical Innovation Center, The Sixth Affiliated Hospital, Sun Yat-sen University, Guangzhou, Guangdong 510655, P. R. China; Cyrus Tang Medical Institute, Collaborative Innovation Center of Hematology, State Key Laboratory of Radiation Medicine and Prevention, Soochow University, Suzhou, Jiangsu 215123, P. R. China; Department of Anaesthesia, The Sixth Affiliated Hospital, Sun Yat-sen University, Guangzhou, Guangdong 510655, P. R. China; Biomedical Innovation Center, The Sixth Affiliated Hospital, Sun Yat-sen University, Guangzhou, Guangdong 510655, P. R. China; Department of Anaesthesia, The Sixth Affiliated Hospital, Sun Yat-sen University, Guangzhou, Guangdong 510655, P. R. China; Biomedical Innovation Center, The Sixth Affiliated Hospital, Sun Yat-sen University, Guangzhou, Guangdong 510655, P. R. China; Department of Anaesthesiology of Centro Hospitalar Conde de Sao Januario, Macao Health Bureau, Macau S.A.R. 999078, P. R. China; Cyrus Tang Medical Institute, Collaborative Innovation Center of Hematology, State Key Laboratory of Radiation Medicine and Prevention, Soochow University, Suzhou, Jiangsu 215123, P. R. China; Department of Radiotherapy, The First Affiliated Hospital of Soochow University, Suzhou, Jiangsu 215006, P. R. China; Cyrus Tang Medical Institute, Collaborative Innovation Center of Hematology, State Key Laboratory of Radiation Medicine and Prevention, Soochow University, Suzhou, Jiangsu 215123, P. R. China; Department of Radiotherapy, The First Affiliated Hospital of Soochow University, Suzhou, Jiangsu 215006, P. R. China; Cyrus Tang Medical Institute, Collaborative Innovation Center of Hematology, State Key Laboratory of Radiation Medicine and Prevention, Soochow University, Suzhou, Jiangsu 215123, P. R. China; Cyrus Tang Medical Institute, Collaborative Innovation Center of Hematology, State Key Laboratory of Radiation Medicine and Prevention, Soochow University, Suzhou, Jiangsu 215123, P. R. China; Department of Anaesthesia, The Sixth Affiliated Hospital, Sun Yat-sen University, Guangzhou, Guangdong 510655, P. R. China; Biomedical Innovation Center, The Sixth Affiliated Hospital, Sun Yat-sen University, Guangzhou, Guangdong 510655, P. R. China

**Keywords:** colorectal cancer, celecoxib, epithelial–mesenchymal transition, propionyl–CoA carboxylase, placental growth factor

## Abstract

**Background:**

Celecoxib is widely used in the prevention and treatment of colorectal cancer (CRC). Although its mechanism of action involves both cyclooxygenase-2 (COX-2)-dependent and COX-2-independent pathways, the non-COX-2 targets of celecoxib remain poorly understood. Preliminary experiments revealed that celecoxib can inhibit the expression of the propionyl–CoA carboxylase alpha chain (PCCA); hence, this study aimed to investigate the role and underlying mechanisms of PCCA as a non-COX-2 target of celecoxib.

**Methods:**

Wound-healing, transwell, and Cell Counting Kit-8 assays were conducted to evaluate the effects of celecoxib on migration, invasion, and proliferation in PCCA-overexpressing CRC cell lines. Western blotting was performed to assess the expression of epithelial–mesenchymal transition (EMT) markers. *In vivo* tumor growth and angiogenesis were examined by using mouse models. Quantitative polymerase chain reaction was used to analyse the transcriptional levels of placental growth factor (*PLGF*).

**Results:**

Celecoxib inhibited PCCA expression in CRC and suppressed PCCA-mediated migration, invasion, and proliferation in COX-2-deficient CRC cell lines (HCT116 and DLD1). These effects were associated with the reversal of PCCA-induced downregulation of E-cadherin and upregulation of N-cadherin and vimentin. In addition, celecoxib inhibited PCCA-driven tumor growth and angiogenesis in HCT116 cells, which correlated with altered *PLGF* expression.

**Conclusion:**

Celecoxib suppresses PCCA-induced EMT and angiogenesis via a COX-2-independent mechanism. These findings suggest that celecoxib may offer additional therapeutic benefits for patients with CRC with elevated PCCA expression and reveal a novel potential antitumor mechanism of celecoxib.

## Introduction

Colorectal cancer (CRC) remains a major global health challenge, ranking as the third-most commonly diagnosed cancer and second-leading cause of cancer-related mortality worldwide [1]. Annually, CRC claims >900,000 lives and its incidence continues to rise, even among populations traditionally considered at low risk [2]. The global burden of CRC is projected to reach 3.2 million new cases and 1.6 million deaths annually by 2040 [3, 4], highlighting the urgent need for continued research.

It is well established that inflammation plays a critical role in the development and progression of CRC [5–7], leading to the hypothesis that non-steroidal anti-inflammatory drugs (NSAIDs) may have therapeutic potential in CRC management [8]. Celecoxib, a widely used NSAID, has been shown to improve CRC prognosis [9–12]. Although initially thought to exert its effects solely through cyclooxygenase-2 (COX-2) inhibition, recent studies have revealed additional non-COX-2 targets [13–15]. The identification of these targets has facilitated both a reduction in celecoxib-associated side effects and the development of novel clinical applications, including its use as an adjuvant therapy [10, 14, 16].

In our previous study, we found that the propionyl–CoA carboxylase alpha chain (PCCA) is highly expressed in CRC and promotes tumor progression, metastasis, and epithelial–mesenchymal transition (EMT) [17], suggesting that PCCA may represent a viable therapeutic target. Preliminary bioinformatics analyses further indicated that celecoxib treatment reduces PCCA expression in CRC tumor tissues. Based on these findings, the present study aimed to investigate the interaction between PCCA and celecoxib, and elucidate the underlying mechanisms by using two COX-2-deficient CRC cell lines as primary models.

In this study, we utilized two COX-2-deficient CRC cell lines as our primary models. Through a series of *in vitro* and *in vivo* experiments, we demonstrated that celecoxib suppresses the PCCA-mediated promotion of EMT and angiogenesis in CRC cells—effects that were associated with changes in placental growth factor (*PLGF*) expression.

These findings offer insights into the therapeutic potential of targeting PCCA in CRC and suggest novel, COX-2-independent mechanisms of action for celecoxib. This work may inform future research into the clinical application of both celecoxib and PCCA as components of CRC therapy.

## Materials and methods

### Cell lines

CRC cell lines (HT29, SW480, DLD1, and RKO) were kindly provided by Dr Ping Lan (Sixth Affiliated Hospital of Sun Yat-sen University, Guangzhou, China). HCT116 cells were obtained from the National Collection of Authenticated Cell Cultures and human embryonic kidney 293T **(**HEK 293T) cell lines were obtained from the American Type Culture Collection (CRL-3216; Manassas, VA, USA).

Cells were cultured in complete medium supplemented with 10% fetal bovine serum (FBS; Cat# SV30208.02; Hyclone, Logan, UT, USA) and 1% penicillin/streptomycin (Cat# 15070063; Gibco, Grand Island, NY, USA). Specifically, HT29, DLD1, and HEK 293T cells were maintained in Dulbecco’s Modified Eagle’s Medium (Cat# C11995500; Gibco), RKO and SW480 cells in Roswell Park Memorial Institute 1640 medium (Cat# C22400500; Gibco), and HCT116 cells in MycCoy’s 5A medium (Cat# C3020-0500; VivaCell, Shanghai, China). All cells were maintained at 37°C in a humidified 5% carbon dioxide incubator (Thermo Scientific, Waltham, MA, USA) and subcultured by using 0.05% trypsin-ethylenediaminetetraacetic acid (Cat#25300054; Gibco).

### Data acquisition

The GSE11237 datasets were retrieved from the Gene Expression Omnibus (GEO) (https://www.ncbi.nlm.nih.gov/geo/) [18]. These datasets were generated by using the Affymetrix HT HG-U133+PM Array Plate and analysed by using R software (version 4.1.1).

### Tumor models

Female nude mice (6–8 weeks old) were obtained from the Changzhou Cavens Experimental Animal Center (Changzhou, China) and housed under specific pathogen-free conditions at Soochow University. All animal procedures were approved by the Soochow University Institutional Animal Care and Ethical Committee (SUDA20240824A02) guidelines. For tumor-growth studies, 2 × 10^6^ HCT116 cells were subcutaneously injected into the flanks of the mice. The tumor dimensions were measured every 3 days by using a vernier caliper and the volume was calculated as: volume = 0.5 × (width^2^ × length). Each group consisted of eight mice and experiments were repeated twice.

### Drug experiments

For *in vitro* treatments, human CRC cell lines (HT29, SW480, RKO, DLD1, and HCT116) were treated with 0, 10, 20, 40, 60, 80, and 100 µM celecoxib (CAS 169590-42-5; Pfizer, New York, NY, USA), dissolved in dimethylsulfoxide (Cat# D2650; Sigma-Aldrich, St. Louis, MO, USA) for 24 or 48 h to assess the dose- and time-dependent effects. For Western blot analysis, cells were treated with 60 µM celecoxib. For wound-healing, transwell migration and invasion, proliferation, and colony-formation assays, cells were treated with 20 µM celecoxib.

For *in vivo* experiments, celecoxib was dissolved in double-distilled water and administered via oral gavage at 3 mg/kg body weight once daily, starting on Day 6 after tumor inoculation. Control mice received an equivalent volume of double-distilled water.

### CCK-8 viability assay

Cell viability was assessed by using the Cell Counting Kit-8 (CCK-8) assay (Cat# FD3788; Fdbio Science, Hangzhou, China). Cells were seeded in 96-well plates at a density of 2 × 10³ cells per well and cultured for 24, 48, 96, 108, and 120 h. At each time point, 10% CCK-8 solution was added to each well and the absorbance was measured at 450 nm after 1 h of incubation at 37°C. Experiments included three technical replicates and were performed in triplicate.

### Western blot

Cells were harvested and lysed in cell lysis buffer (Cat# P0013; Beyotime, Shanghai, China) supplemented with 1% phenylmethanesulfonyl fluoride (Cat#ST507; Beyotime). The protein concentration was determined by using a bicinchoninic acid kit (Cat#P0010; Beyotime). Proteins were separated on 10% or 12% sodium dodecyl sulfate–polyacrylamide gel electrophoresis gels (Cat#PG112; Epizyme, Shanghai, China) and subsequently transferred onto polyvinylidene fluoride membranes (Cat# IPVH00010; Merck Millipore, Billerica, MA, USA). Following blocking with Protein-Free Rapid Blocking Buffer (Cat#PS108; Epizyme), membranes were incubated overnight at 4°C with primary antibodies (Supplementary Table S1). After washing, membranes were incubated for 30 min at room temperature with DyLight 800 4X polyethylene glycol-conjugated IgG (H + L) antibodies (1:10,000; anti-rabbit, Cat# 5151; anti-mouse, Cat#5257; Cell Signaling Technology, Danvers, MA, USA). Blots were visualized by using a two-color infrared imaging system. Experiments were repeated three times, with representative blots shown.

### cDNA and lentivirus infection

The PCCA cDNA (Tsingke Biotechnology, Beijing, China) was transiently transfected into cells by using Lipo8000 (Cat#C0533; Beyotime). For stable transfection, lentiviruses were generated by co-transfecting the PCCA cDNA with two packaging plasmids (psPAX2 and pMD2.G; Tsingke Biotechnology) into HEK 293T cells using Lipofectamine 3000 (Cat#L3000008; ThermoFisher, Waltham, MA, USA). After 48 h, the supernatants were harvested and used to infect HCT116 cells in the presence of polybrene (2 µg/mL). Stable cell lines were selected by using puromycin (2 mg/mL; Cat# ST551; Beyotime).

### Wound-healing assay

Cells (2 × 10^6^/well) were seeded in six-well plates that were pre-marked for consistent wound placement. Upon attaining ∼90% confluence, a 200-µL pipette tip was used to create uniform scratches. Detached cells were removed by washing with phosphate-buffered saline (PBS) and fresh medium was added. Migration was monitored at 0, 24, and 48 h by using an inverted microscope and quantified by using ImageJ software (v1.48; NIH). Experiments were performed in triplicate and repeated three times.

### Transwell migration and invasion assays

Transwell migration and invasion assays were performed by using a Boyden chamber (Cat# 725421; NEST, Jiangsu, China) with an 8-µm pore size in 24-well plates, with or without Matrigel (Cat# 356231; Corning Incorporated, Corning, NY, USA). Cells (1 × 10^5^) suspended in 200 µL of medium containing 1% FBS were added to the upper chamber while the lower chamber contained 600 µL of medium with 10% FBS. After 24 h, cells were fixed with 4% paraformaldehyde (PFA), stained with 0.1% crystal violet solution (Cat# C0121; Beyotime) diluted at 1:10 in PBS, and counted in three random fields per insert. Each experiment was repeated three times.

### Colony-formation assay

Cells were seeded into six-well plates at a density of 500 cells/well and incubated for 7 days. After incubation, cells were fixed with 4% PFA and stained with 0.1% crystal violet solution (Cat# C0121; Beyotime) diluted at 1:10 in PBS. Colonies containing >50 cells in three randomly selected fields were counted. The experiment was repeated three times.

### Quantitative reverse-transcription polymerase chain reaction

Quantitative reverse-transcription polymerase chain reaction was performed as previously described [19]. Briefly, total RNA from tissues was isolated by using an RNA freeze reagent (Cat# R711-01; Vazyme, Jiangsu, China), while cellular RNA was isolated by using the RNA Isolation Kit (Cat# RC112-01; Vazyme). RNA quality and quantity were measured by using a NanoDrop spectrophotometer (Cat# ND-2000; Thermo Scientific). RNA was reverse transcribed into complementary DNA by using the HiScript III RT SuperMix for qPCR (+ gDNA wiper) (Cat# R323-01; Vazyme). Real-time qPCR was performed by using a Roche LightCycler 480 and ChamQ SYBR Color qPCR Master Mix (without ROX) (Cat# Q4421-02; Vazyme). The relative gene expression was calculated by using the 2−ΔΔCT method and normalized to *B-actin*. The primers used for qPCR are listed in Supplementary Table S2.

### Immunofluorescence

Tumor blood vessel staining and analysis were performed as previously described [20]. Briefly, the mice were intravenously (*i.v.*) injected with Hoechst 33342 (10 mg/kg in 200 µL of PBS; Cat#B2261; Sigma-Aldrich). After 5 min, the mice were perfused with PBS and tumor tissues were isolated, fixed in 4% PFA at 4°C for 3 h, and then incubated overnight in 30% sucrose at 4°C. The tissues were embedded in optical cutting temperature compound (Cat#4583; Sakura Finetek, Torrance, CA, USA) and stored at −80°C. Frozen samples were sectioned at a thickness of 20 µm. Tumor sections were incubated overnight at 4°C with primary antibodies against CD31 (1:200; clone MEC13.3; Cat# 550274; BD Biosciences, San Jose, CA, USA) followed by incubation with Alexa Fluor 647 Goat Anti-Armenian Hamster secondary antibody (1:200; Cat# 127–605-160; Jackson ImmunoResearch, West Grove, PA, USA) for 2 h at 20°C–25°C in humid chambers in the dark. Sections were counterstained with Sytox Green. The fluorescence intensities of CD31 and Hoechst 33342, measured in four to six randomly selected fields per tumor tissue, were used to calculate the mean fluorescence intensity, reflecting vessel density (CD31) and vessel perfusion (Hoechst 33342). Images were captured by using an Olympus FV3000 confocal laser-scanning microscope (Tokyo, Japan) and the vessel density was calculated by using Image-Pro Plus software (version 6.0). Each experiment was repeated twice.

### Statistical analysis

Statistical analyses were performed by using SPSS Statistics (version 19.0; SPSS Inc., Chicago, IL, USA) and GraphPad Prism (version 9.0). For two-group comparisons, normally distributed data were analysed by using the independent samples *t*-test and non-normally distributed data were analysed by using the Mann–Whitney *U* test. For multi-group comparisons, one-way analysis of variance (ANOVA) with the Turkey’s multiple comparison test was used. For two categorical variables with quantitative outcomes, two-way ANOVA was applied. Statistical significance was defined as a two-tailed *P* value of <0.05. Data are presented as mean ± standard error of mean for normally distributed data or median ± interquartile range for non-normally distributed data, unless otherwise specified.

## Results

### Celecoxib inhibits CRC cell growth and suppresses PCCA expression

**Figure 1:**
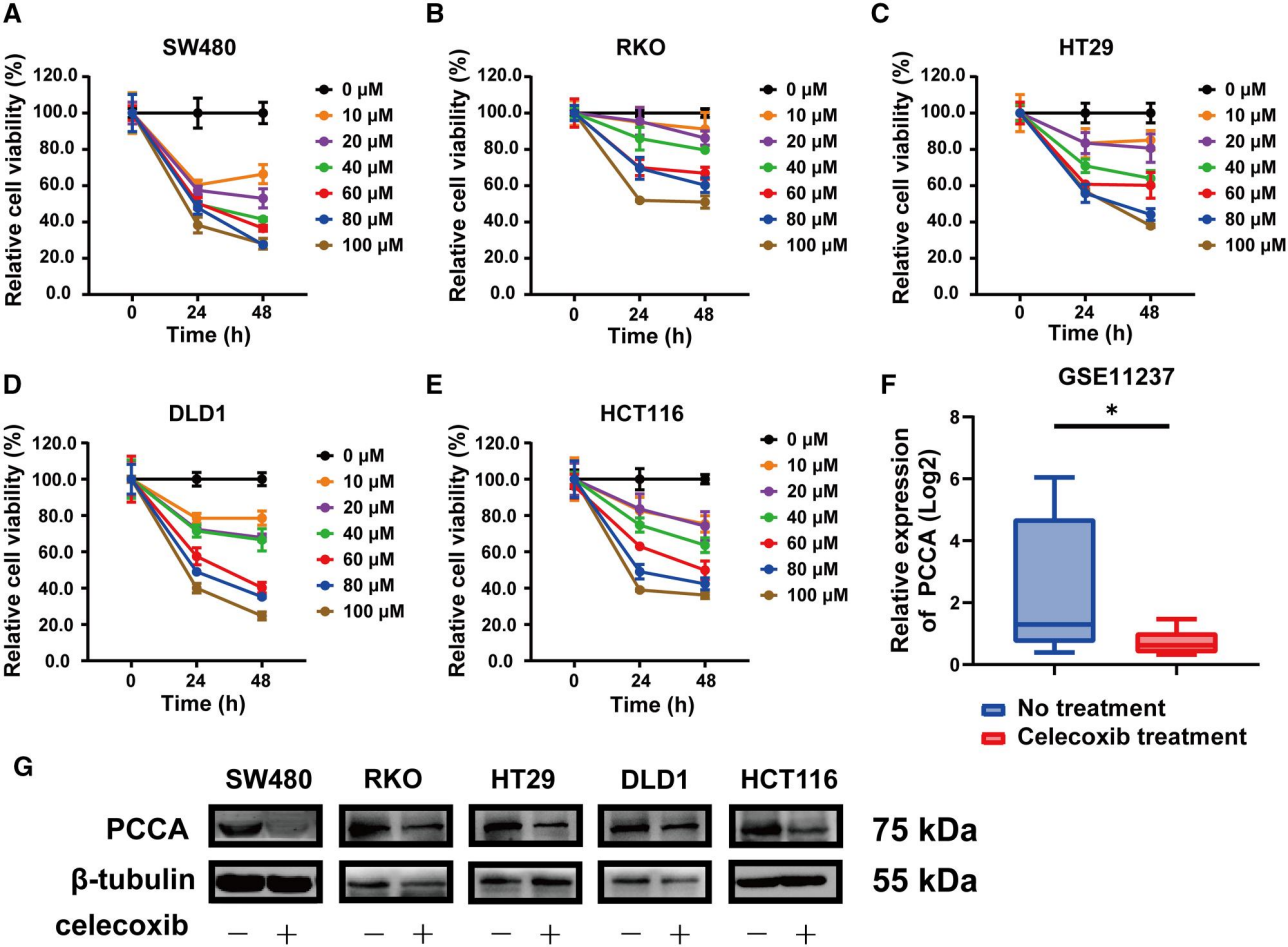
Celecoxib inhibits CRC cell growth and suppresses PCCA expression. (A–E) CCK-8 assays were used to assess the proliferation of SW480, HT29, RKO, DLD1, and HCT116 CRC cell lines treated with varying concentrations of celecoxib (0, 10, 20, 40, 60, 80, and 100 µM) for 24 or 48 h (*n =* 3). (F) Box plot showing PCCA gene expression in tumor tissues from patients with (*n =* 11) or without (*n =* 12) celecoxib treatment for 7 days before surgery (dates were from the GEO dataset: GSE11237). (G) Representative Western blot images of PCCA protein expression in SW480, HT29, RKO, DLD1, and HCT116 cells treated with or without 60 µM celecoxib for 24 h. Data are from one experiment representative of three independent experiments with similar results. 0–100 µM: celecoxib-treated groups with varying concentrations. No treatment: tumor tissues from patients not treated with celecoxib (*n =* 12). Celecoxib treatment: tumor tissues from patients treated with celecoxib (400 mg, BID) for 7 days before surgery (*n =* 11). Unpaired Student’s *t*-tests were performed to assess statistical significance. Data are presented as mean ± SEM (*P < 0.05). GEO = Gene Expression Omnibus, µM = micromolar, BID = twice daily, SEM = standard error of the mean.

To evaluate the effects of celecoxib on CRC cells, five CRC cell lines—SW480, RKO, HT29, DLD1, and HCT116—were treated with varying concentrations of celecoxib (0, 10, 20, 40, 60, 80, and 100 µM) for either 24 or 48 h (Fig. 1A–E). The results demonstrated that celecoxib inhibited cell growth in a dose- and time-dependent manner. Based on these findings, 60 µM for 24 h was selected as the optimal treatment condition for subsequent Western blot analysis.

Furthermore, analysis of the GSE11237 dataset from the GEO database revealed a significant reduction in PCCA expression in CRC tumor tissues following celecoxib treatment (Fig. 1F). This decrease in PCCA expression was further confirmed in CRC cell lines (Fig. 1G).

### Celecoxib suppresses the pro-EMT effect of PCCA in COX-2-deficient CRC cell lines

**Figure 2:**
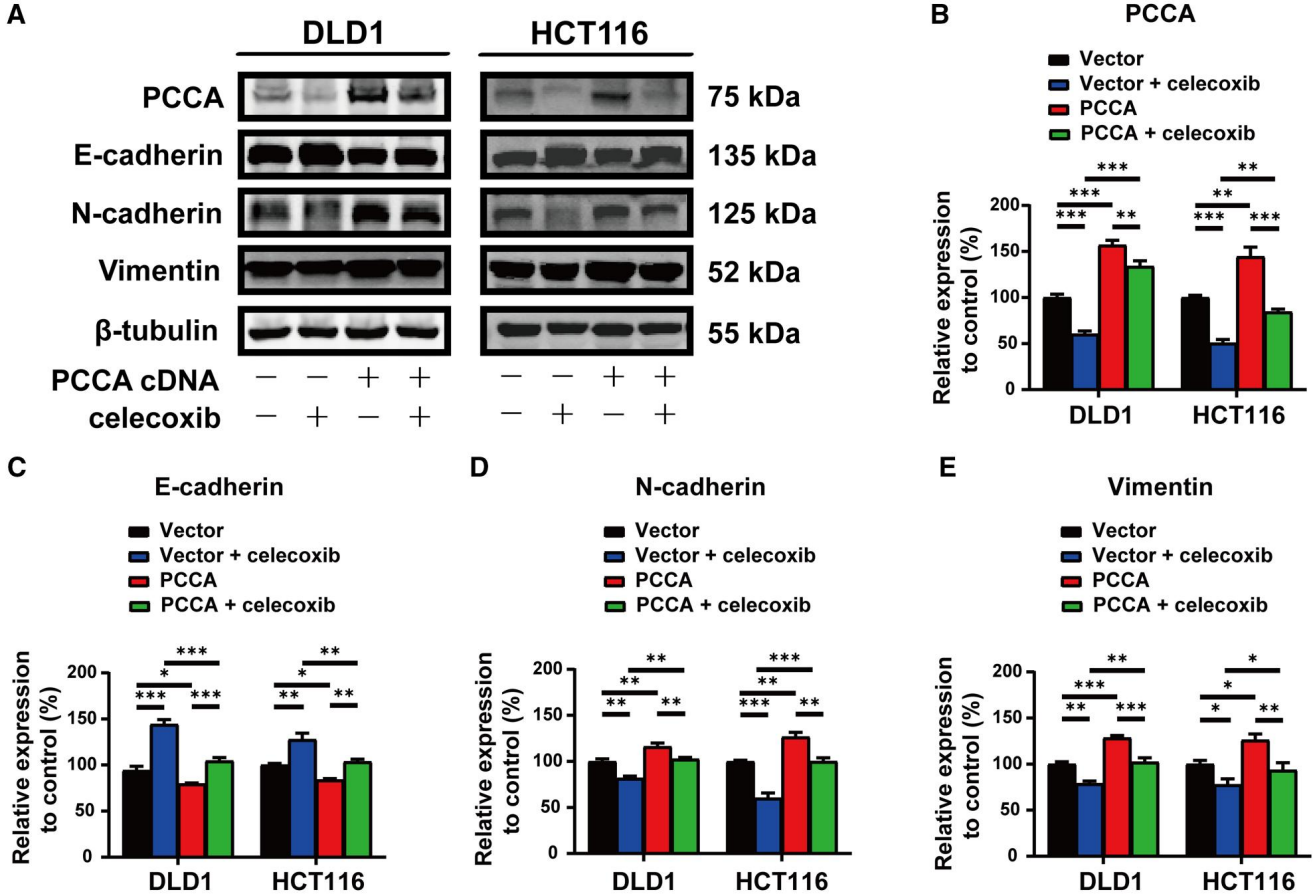
Celecoxib suppresses the pro-EMT effect of PCCA in COX-2-deficient CRC cell lines. (A–E) Representative Western blot images and corresponding quantification of PCCA, the epithelial marker E-cadherin, and the mesenchymal markers N-cadherin and vimentin in DLD1 and HCT116 cells transfected with either PCCA cDNA or a control vector plasmid, with (60 µM celecoxib for 24 h) or without (0 µM celecoxib for 24 h) celecoxib treatment. Data are from one experiment representative of three independent experiments with similar results. Vector: CRC cells transfected with the control vector plasmid and treated with 0 µM celecoxib for 24 h. Vector + celecoxib: CRC cells transfected with the control vector plasmid and treated with 60 µM celecoxib for 24 h. PCCA: CRC cells transfected with PCCA cDNA plasmid and treated with 0 µM celecoxib for 24 h. PCCA + celecoxib: CRC cells transfected with PCCA cDNA plasmid and treated with 60 µM celecoxib for 24 h. Statistical comparisons were performed by using one-way ANOVA with Tukey’s post-hoc test. Data are presented as mean ± SEM (*P < 0.05, **P < 0.01, ***P < 0.001). µM = micromolar, SEM = standard error of the mean.

The anti-EMT effect of celecoxib through COX-2 inhibition is well established [21, 22]. To eliminate COX-2-related interference, we employed COX-2-deficient CRC cell lines DLD1 and HCT116. As expected, PCCA overexpression significantly decreased the epithelial marker E-cadherin and increased mesenchymal markers such as N-cadherin and vimentin. Celecoxib treatment reversed these changes, upregulating epithelial marker expression and downregulating mesenchymal marker expression in both vector control and PCCA-overexpressing groups (Fig. 2).

### Celecoxib inhibits PCCA-mediated migration and invasion in COX-2-deficient CRC cell lines

**Figure 3:**
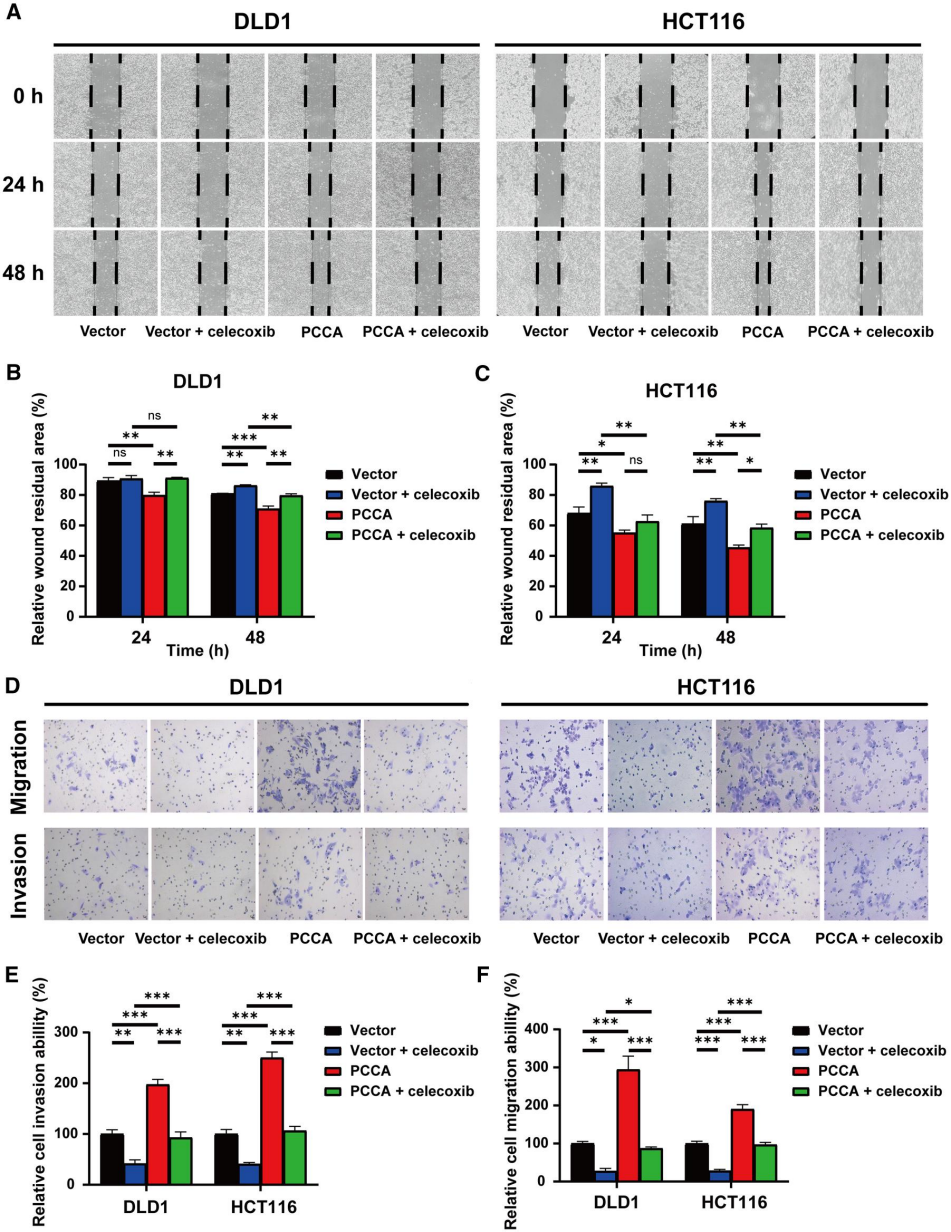
Celecoxib inhibits PCCA-mediated migration and invasion in COX-2-deficient CRC cell lines. (A–C) Wound-healing assays were conducted on DLD1 and HCT116 cells transfected with PCCA cDNA or vector plasmid, with (20 µM) or without (0 µM) celecoxib treatment at the indicated time points (0, 24, and 48 h). (D–F) Representative images and quantification of transwell migration and invasion assays in DLD1 and HCT116 cells transfected with PCCA cDNA or vector plasmid, with (20 µM) or without (0 µM) celecoxib treatment. Data are from one experiment representative of three independent experiments with similar results, each of which included three technical replicates and was performed twice. Vector: CRC cells transfected with the vector plasmid and treated with 0 µM celecoxib. Vector + celecoxib: CRC cells transfected with the vector plasmid and treated with 20 µM celecoxib. PCCA: CRC cells transfected with PCCA cDNA plasmid and treated with 0 µM celecoxib. PCCA + celecoxib: CRC cells transfected with PCCA cDNA plasmid and treated with 20 µM celecoxib. Statistical comparisons were performed by using one-way ANOVA with Tukey’s post-hoc test. Data are presented as mean ± SEM (*P < 0.05, **P < 0.01, ***P < 0.001). µM = micromolar, SEM = standard error of the mean.

Wound-healing (Fig. 3A–C) and transwell migration and invasion assays (Fig. 3D–F) demonstrated that PCCA overexpression significantly enhanced the migratory and invasive capacities of the COX-2-deficient CRC cell lines DLD1 and HCT116. Notably, celecoxib treatment reduced these enhanced migratory and invasive behaviors in both vector control and PCCA-overexpressing groups.

### Celecoxib suppresses the PCCA-mediated promotion of proliferation and clonogenicity in COX-2-deficient CRC cell lines

**Figure 4:**
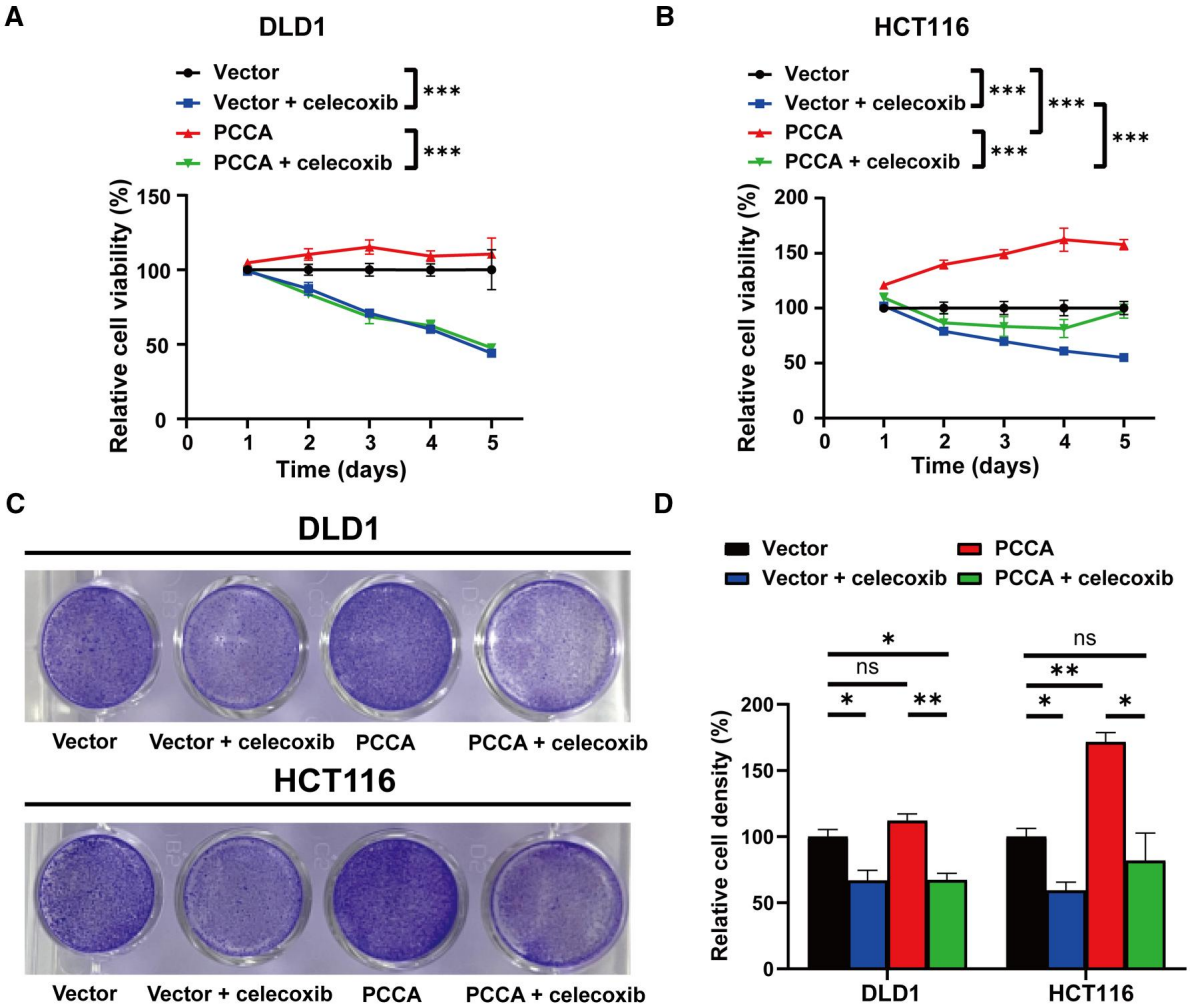
Celecoxib suppresses the promotional effect of PCCA-mediated proliferation and clonogenicity in COX-2-deficient CRC cell lines. (A, B) CCK-8 assays were used for assessing the proliferation of DLD1 and HCT116 cells transfected with PCCA cDNA or vector plasmid, with (20 µM) or without (0 µM) celecoxib treatment. (C, D) Representative images and quantification of colony formation in DLD1 and HCT116 cells transfected with PCCA cDNA or vector plasmid, with (20 µM) or without (0 µM) celecoxib treatment. Data are from one experiment representative of three independent experiments with similar results, each of which included three technical replicates and was performed in triplicate. Vector: CRC cells transfected with the vector plasmid and treated with 0 µM celecoxib. Vector + celecoxib: CRC cells transfected with the vector plasmid and treated with 20 µM celecoxib. PCCA: CRC cells transfected with PCCA cDNA plasmid and treated with 0 µM celecoxib. PCCA + celecoxib: CRC cells transfected with PCCA cDNA plasmid and treated with 20 µM celecoxib. Statistical comparisons were performed by using (c, d) one-way ANOVA or (a, b) two-way ANOVA with Tukey’s post-hoc test. Data are presented as mean ± SEM (*P < 0.05, **P < 0.01, ***P < 0.001). µM = micromolar, SEM = standard error of the mean.

To investigate the effects of celecoxib on cellular proliferation and clonogenicity, we assessed HCT116 and DLD1 cells overexpressing PCCA. PCCA overexpression markedly increased proliferation **(**Fig. 4A and B) and colony-forming ability (Fig. 4C and D) in HCT116 cells while the effects were less pronounced in DLD1 cells. Notably, celecoxib treatment significantly suppressed both proliferation and clonogenicity in vector control and PCCA-overexpressing groups, although its inhibitory effect on HCT116 cell growth was less pronounced than that observed in DLD1 cells.

### Celecoxib suppresses PCCA-mediated tumor growth and angiogenesis in HCT116 cells

**Figure 5:**
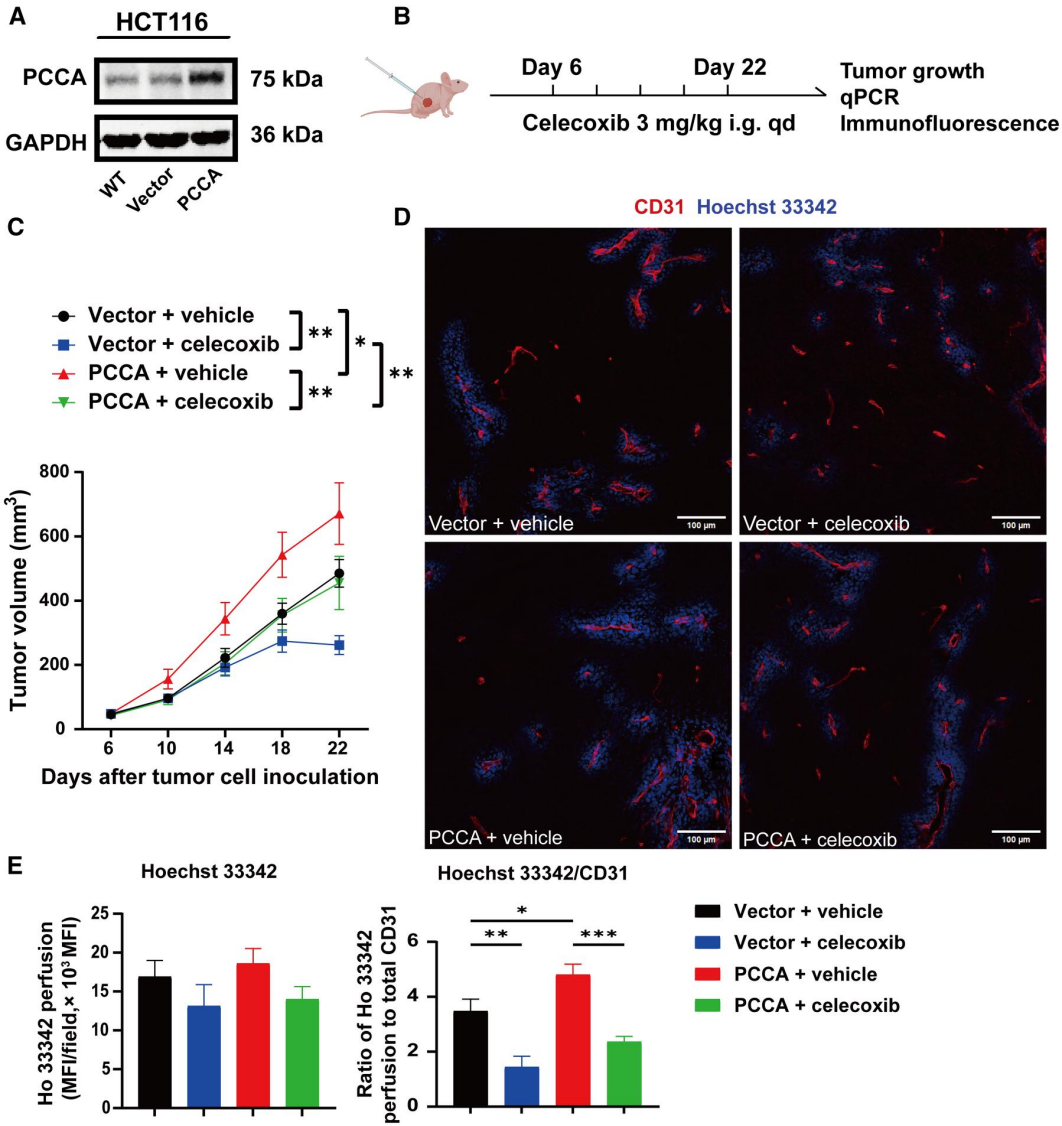
Celecoxib suppresses PCCA-mediated tumor growth and angiogenesis in HCT116 cells. (A) Representative Western blot images showing PCCA expression in HCT116-WT, HCT116-Vector cells, or HCT116-PCCA cells. (B) Diagram of subcutaneous tumor models. (C) Tumor-growth curves of HCT116-PCCA or vector cells in nude mice, with (3 mg/kg, intragastrically, once daily) or without (equivalent volume of water via intragastric gavage) celecoxib treatment (*n =* 8). (D) Representative images of immunofluorescence showed the tumor vessel perfusion (Hoechst 33342, nuclear perfusion) and vessel density (CD31, endothelial cell staining) of the tumor tissue. Scale bar = 100 µm (*n =* 6). (E) Quantification of Hoechst 33342-perfused tumor areas and the proportion of perfused area (Hoechst 33342-positive area) relative to the total vessel density (CD31-positive area) (*n =* 6). Data are from one experiment representative of (C, D) two or (A) three independent experiments with similar results. WT: parental HCT116 cell line. Vector: stable HCT116 cell line carrying the vector plasmid. PCCA: stable HCT116 cell line carrying the PCCA cDNA plasmid. Vector + vehicle: mice inoculated with stable HCT116 vector cells and administered an equivalent volume of water via intragastric gavage. Vector + celecoxib: mice inoculated with stable HCT116 vector cells and treated with celecoxib (3 mg/kg, intragastrically, once daily). PCCA + vehicle: mice inoculated with stable HCT116 PCCA cells and administered an equivalent volume of water via intragastric gavage. PCCA + celecoxib: mice inoculated with stable HCT116 PCCA cells and treated with celecoxib (3 mg/kg, intragastrically, once daily). Statistical comparisons were performed by using (E) one-way ANOVA or (C) two-way ANOVA with Tukey’s post-hoc test. Data are presented as mean ± SEM (*P < 0.05, **P < 0.01, ***P < 0.001). SEM = standard error of the mean.

Our findings indicate that celecoxib may inhibit PCCA-driven effects in CRC cell lines, with particularly strong effects observed in HCT116 cells. To investigate this further, we established stable PCCA-overexpressing HCT116 cells by using a lentiviral system (Fig. 5A). As expected, PCCA overexpression promoted tumor growth whereas celecoxib treatment significantly suppressed tumor growth in both vector control and PCCA-overexpressing groups (Fig. 5B and C).

In our previous study, we observed central necrosis in tumors from control mice during late-stage growth—a feature absent in tumors overexpressing PCCA [17]. Given the established link between EMT and tumor angiogenesis, and the inherently high invasiveness of HCT116 cells, we explored whether the PCCA-mediated effects were associated with enhanced angiogenesis. We hypothesized that the absence of central necrosis could be attributed to accelerated tumor growth and increased angiogenesis.

To evaluate this *in vivo*, we examined tumor angiogenesis by using a functional vascular labeling method. Hoechst 33342 dye was intravenously injected 5 min prior to tumor harvest to label perfused blood vessels. Immunofluorescence analysis revealed a higher Hoechst 33342/CD31 signal ratio in PCCA-overexpressing tumors, indicating increased vascular perfusion (Fig. 5D and E). In contrast, PCCA knockdown resulted in reduced vascular perfusion (Supplementary Figure S1C and D). Notably, celecoxib treatment diminished the elevated perfusion observed in PCCA-overexpressing tumors, suggesting that celecoxib inhibits PCCA-mediated angiogenesis (Fig. 5D and E).

### *PLGF* as a potential mediator of the interaction between celecoxib and PCCA

**Figure 6:**
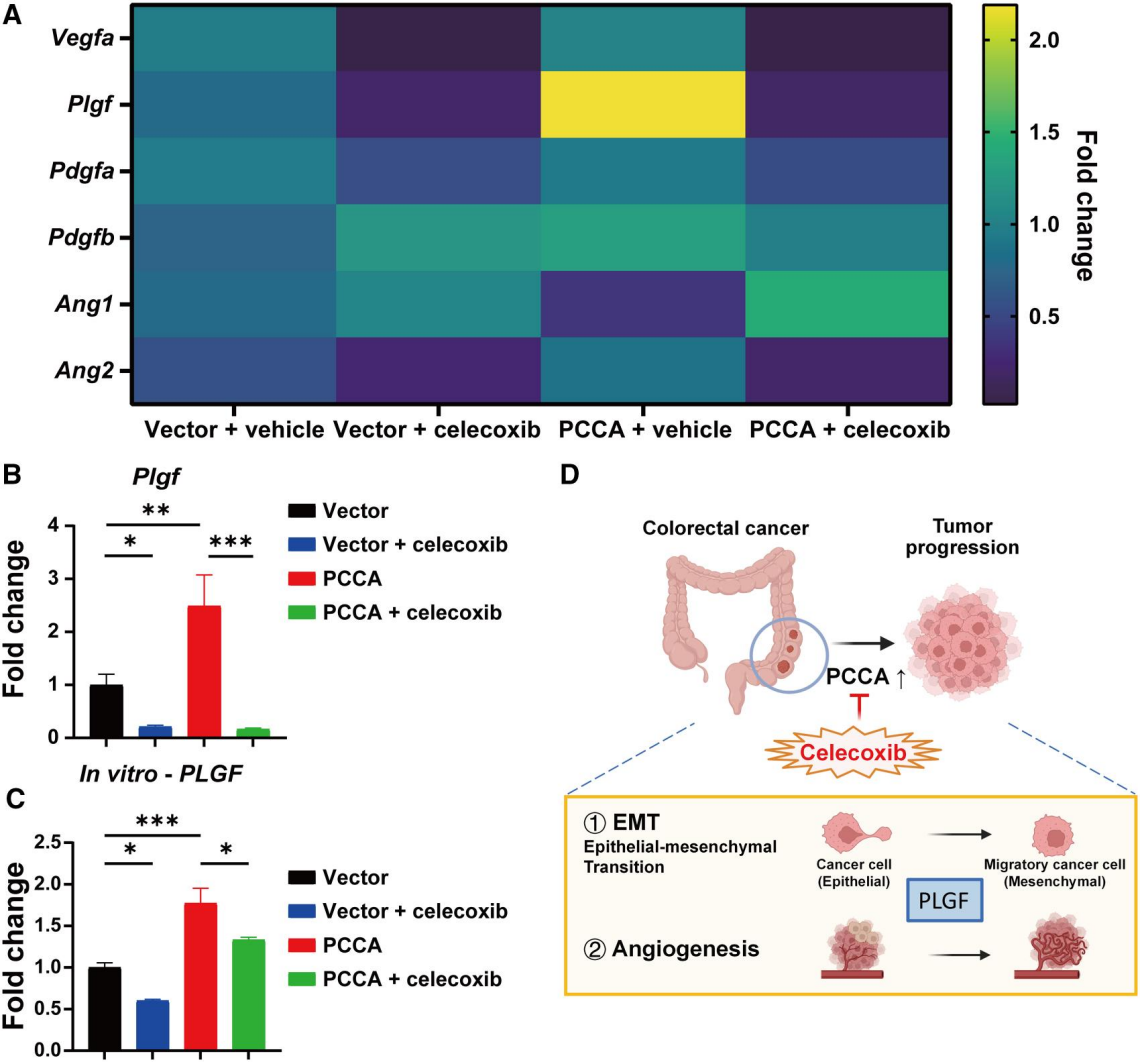
*PLGF* as a potential mediator of the interaction between celecoxib and PCCA. (A) Heat map showing the transcription levels assessed by using qPCR of angiogenesis-related marker genes in tumor tissues of HCT116-PCCA and vector groups, with (3 mg/kg celecoxib, intragastrically, once daily) or without (an equivalent volume of water via intragastric gavage) celecoxib treatment (*n =* 6). (B) qPCR showed the transcription levels of *Plgf* in tumor tissues of HCT116-PCCA or vector cells, with (60 µM) or without (0 µM) celecoxib treatment (*n =* 6). (C) qPCR showed the transcription levels of *PLGF* in HCT116-PCCA and vector cell lines, with or without celecoxib treatment (*n =* 4). (D) Summary of this research showing that celecoxib inhibits the PCCA-mediated promotion of CRC EMT and angiogenesis, which is related to *PLGF*. Created with BioRender.com (2025). Vector + vehicle: mice inoculated with a stable HCT116 cell line carrying the vector plasmid and administered an equivalent volume of water via intragastric gavage. Vector + celecoxib: mice inoculated with a stable HCT116 cell line carrying the vector plasmid and treated with celecoxib (3 mg/kg, intragastrically, once daily). PCCA + vehicle: mice inoculated with a stable HCT116 cell line carrying the PCCA cDNA plasmid and administered an equivalent volume of water via intragastric gavage. PCCA + celecoxib: mice inoculated with a stable HCT116 cell line carrying the PCCA cDNA plasmid and treated with celecoxib (3 mg/kg, intragastrically, once daily). Vector: a stable HCT116 cell line carrying the vector plasmid and treated with 0 µM celecoxib. Vector + celecoxib: a stable HCT116 cell line carrying the vector plasmid and treated with 60 µM celecoxib. PCCA: a stable HCT116 cell line carrying the PCCA cDNA plasmid and treated with 0 µM celecoxib. PCCA + celecoxib: a stable HCT116 cell line carrying the PCCA cDNA plasmid and treated with 60 µM celecoxib. Statistical comparisons were performed by using one-way ANOVA with Tukey’s post-hoc test. Data are presented as mean ± SEM (*P < 0.05, **P < 0.01, ***P < 0.001). SEM = standard error of the mean.

To identify key mediators underlying the interaction between celecoxib and PCCA, we examined the transcriptional levels of several angiogenesis-related genes known to contribute to both tumor angiogenesis and EMT in murine *in vivo* models [23–28], including vascular endothelial growth factor alpha (*Vegfa*), *Plgf*, platelet-derived growth factor alpha polypeptide (*Pdgfa*), platelet-derived growth factor subunit B (*Pdgfb*), Angiopoietin-1 (*Ang1*), and Angiopoietin-2 (*Ang2*). Celecoxib treatment significantly attenuated the PCCA-induced upregulation of these genes, with particularly notable suppression of *Plgf* levels (Fig. 6A and B). Furthermore, PCCA knockdown resulted in decreased *Plgf* levels, reinforcing the role of PCCA in regulating *Plgf* (Supplementary Figure S1E and F).

To further validate this observation and connect it to EMT-related phenotypes observed *in vitro* (Figs 2–4), we assessed the human-derived *PLGF* transcription levels in HCT116 cells. As expected, celecoxib significantly reduced PCCA-induced *PLGF* upregulation (Fig. 6C) while PCCA knockdown led to a corresponding decrease in *PLGF* expression (Supplementary Figure S1G).

In summary, celecoxib suppresses PCCA-driven CRC progression by inhibiting both EMT and angiogenesis, with *PLGF* potentially serving as a critical mediator of this process (Fig. 6D).

## Discussion

CRC remains a growing global health burden [1]. Celecoxib, a selective COX-2 inhibitor, has been widely used in the prevention and treatment of colorectal tumors, with generally favorable outcomes [9–12]. In this study, we extended our previous research on novel molecular targets in CRC and identified PCCA as a non-COX-2 target of celecoxib. This finding opens up new avenues for therapeutic strategies, such as using celecoxib as an adjunct treatment in patients with elevated PCCA expression. In addition, this discovery supports the development of celecoxib derivatives lacking COX-2 activity to minimize the associated side effects.

Lipid metabolic reprogramming plays a pivotal role in tumor progression [29–31], as highly proliferative tumor cells are particularly dependent on lipid metabolism [32, 33]. PCCA, an enzyme involved in the catabolism of odd-chain fatty acids and branched-chain amino acids, has been implicated in CRC progression [17, 34, 35]. Notably, celecoxib has been shown to downregulate various lipid metabolism-related genes [18, 36–38]. In a clinical trial, transcriptomic profiling of patients with CRC treated with celecoxib revealed a significant downregulation of >90% of genes involved in lipid metabolism, including PCCA [18]. These findings suggest that the antitumor activity of celecoxib may be partially attributed to the suppression of lipid metabolic pathways.

EMT is a key process in tumor metastasis, stemness, and therapy resistance [39, 40]. Previous studies have shown that celecoxib can inhibit EMT via multiple signaling pathways [21, 41]. Given that EMT is closely associated with tumor angiogenesis, we also examined the anti-angiogenic effects of celecoxib, which have been documented in various tumor models [42–44]. In our *in vivo* experiments, tumors overexpressing PCCA exhibited accelerated growth and reduced central necrosis—findings consistent with those of previous studies [17]. We hypothesized that enhanced angiogenesis might underlie this phenotype. Immunofluorescence analysis confirmed increased functional vasculature in PCCA-overexpressing tumors. Conversely, celecoxib treatment mitigated this vascular enhancement, resulting in greater central necrosis and a “dead volcano” morphology.

We further analysed angiogenesis-related gene expression and found that PLGF—a member of the vascular endothelial growth factor (VEGF) family and a known pro-angiogenic factor [23, 45]—was significantly upregulated in PCCA-overexpressing tumors. This effect was reversed by PCCA knockdown, indicating that PLGF may serve as a downstream mediator of PCCA-driven angiogenesis. The PLGF/vascular endothelial growth factor receptor (VEGFR) pathway is part of the VEGF/VEGFR axis and is well known to be associated with extracellular regulated protein kinases (ERK) phosphorylation [46, 47]. Our previously published work demonstrated that PCCA knockdown affects ERK phosphorylation [17], suggesting that PCCA may influence tumor angiogenesis through modulation of the PLGF/VEGFR/ERK signaling pathway.

Tumor angiogenesis is essential for sustaining tumor growth and enabling metastasis [48]. Like normal tissues, tumors require an adequate vascular supply to meet their metabolic demands [49]. However, tumor vasculature is often disorganized, poorly perfused, and structurally abnormal, lacking proper endothelial support and pericyte coverage [50]. Although some functional vessels can improve drug delivery and immune infiltration [51], others may contribute to therapy resistance. Recent studies have revealed that tumor cells can exploit existing blood vessels through a process known as vascular co-option, enabling tumor growth without inducing new vessel formation. This phenomenon presents a major obstacle to anti-angiogenic therapies, especially in tumors with rich vascular networks [52–56].

Nonetheless, this study has some limitations worth noting. Tumor angiogenesis is intricately linked to the immune microenvironment [57, 58]. Our *in vivo* experiments were conducted in immunodeficient nude mice, which lack a complete immune system. Although this model offers valuable insights, it cannot fully replicate the complexity of the tumor-immune microenvironment in human CRC. This is a known limitation of preclinical studies utilizing human tumor xenografts. Furthermore, we did not perform additional experiments in COX-2-sufficient cell lines, as our aim was to specifically investigate the non-COX-2 effects of PCCA. This approach allowed us to isolate the role of PCCA without introducing confounding interactions with COX-2, in line with the principles of precision medicine. Moreover, our attempts with other cell lines, such as SW480, yielded limited effects, indicating that the monotherapy targeting of PCCA may be insufficient for all CRC types. Consequently, we propose that future strategies should explore combinatorial approaches to enhance treatment efficacy.

In conclusion, our findings demonstrate that celecoxib effectively inhibits PCCA-mediated CRC progression by suppressing both EMT and angiogenesis, likely through the downregulation of *PLGF*. These results highlight the potential clinical value of celecoxib in patients with CRC and elevated PCCA expression, and provide a foundation for developing COX-2-independent celecoxib derivatives with reduced toxicity.

## Supplementary Material

goag071_Supplementary_Data
