# Supplementary material for: Celecoxib suppresses colorectal cancer growth by inhibiting propionyl–CoA carboxylase alpha chain-mediated epithelial–mesenchymal transition and angiogenesis
Source: Gastroenterol Rep (Oxf). 2026 Aug 10;14:goag071. doi: 10.1093/gastro/goag071 (PMC13457091; doi:10.1093/gastro/goag071)
Supplement: Supplementary file 1 [file goag071_supplementary_data.zip › supplementary-figure-2.docx]

**Supplementary Figure S1. PCCA knockdown inhibits tumor angiogenesis and is associated with reduced PLGF level.**

**(A)** Representative Western blot images of COX-2 in RKO, SW480, DLD1, HCT116, and HT29 cell lines. **(B)** Expression levels of the COX-2 gene in RKO, SW480, DLD1, HCT116, and HT29 CRC cell lines obtained from the CCLE database. **(C)** Representative images of immunofluorescence showed the tumor vessel perfusion (Hoechst 33342, in blue) and vessel density (CD31, in red). Scale bar = 50 μm. (n=6). **(D)** Quantification of Hoechst 33342-perfused tumor area and the proportion of perfused area relative to total vessel density (CD31-positive area). (n=6). **(E)** Heatmap displaying the transcriptional levels assessed by qPCR of angiogenesis-related marker genes in tumor tissues from HCT116-PCCA-sh and negative control (NC) groups. (n=7). **(F)** Transcription levels of *Plgf* in tumor tissues of HCT116-PCCA-sh or NC groups. **(G)** Transcription levels of *Plgf* in HCT116-PCCA-sh or NC cell line. NC, mice tumor tissue inoculated with a stable transformation strain of HCT116 with NC RNA. PCCA-sh, mice tumor tissue inoculated with a stable transformation strain of HCT116 with *PCCA* shRNA. Data are presented as Means ± SEM (**P* < 0.05; ** *P* < 0.01; ****P* < 0.001).
