# Supplementary material for: Celecoxib suppresses colorectal cancer growth by inhibiting propionyl–CoA carboxylase alpha chain-mediated epithelial–mesenchymal transition and angiogenesis
Source: Gastroenterol Rep (Oxf). 2026 Aug 10;14:goag071. doi: 10.1093/gastro/goag071 (PMC13457091; doi:10.1093/gastro/goag071)
Supplement: Supplementary file 1 [file goag071_supplementary_data.zip › GR-2025-537.R3_Supplementary-table-2.docx]

**Supplementary Table S1. List of antibodies.**

| **Antigens** | **Species antibodies raised in** | **Dilution**  **(WB)** | **Supplier** | |
| --- | --- | --- | --- | --- |
| PCCA | Rabbit, Polyclonal | 1:3000 | Proteintech, China,  Cat. #21988-1-AP |  |
| CDH1/E-Cadherin | Rabbit, polyclonal | 1:3000 | Boster, China,  Cat. # PB9561 |  |
| CDH2/N-Cadherin | Rabbit, monoclonal | 1:1000 | Boster, China,  Cat. # BM3921 |  |
| Vimentin | Mouse, monoclonal | 1:2000 | Boster, China,  Cat. # BM0135 |  |
| GAPDH | Mouse, monoclonal | 1:2000 | Beyotime, China,  Cat. # AF2819 |  |
| COX2/Cyclooxygenase 2 | Rabbit, monoclonal | 1:1000 | Zenbio, China,  Cat. # AF23971 |  |
| β-tubulin | Mouse, monoclonal | 1:3000 | Beyotime, China,  Cat. #AF2835 |  |

**Supplementary Table S2. List of primers for quantitative PCR.**

| **Gene** | **Primer** | **Sequence (5’-3’)** |
| --- | --- | --- |
| **m*Vegfa*** | **Forward** | **CGGGCCTCGGTTCCAG** |
|  | **Reverse** | **TGAACTTGATCACTTCATGGGACT** |
| **m*Plgf*** | **Forward** | **ATTCAGTCCGTCCTGTGTCC** |
|  | **Reverse** | **GGTTTTCCTCCTTTCTGCCT** |
| **m*Pdgfa*** | **Forward** | **GGAGGAGACAGATGTGAGGTG** |
|  | **Reverse** | **GGAGGAGAACAAAGACCGCA** |
| **m*Pdgfb*** | **Forward** | **TGTGAGACAGTAGTGACCCCT** |
|  | **Reverse** | **AAATAACCCTGCCCACACTCTT** |
| **m*Ang1*** | **Forward** | **ATGGAAAATTATACTCAGTGGCTGC** |
|  | **Reverse** | **ATTTAGTACCTGGGTCTCAACATC** |
| **m*Ang2*** | **Forward** | **TACAAAGAGGGCTTCGGGAG** |
|  | **Reverse** | **GTTGGACTCTTCACCAGCGA** |
| **m*B-actin*** | **Forward** | **ATCGTGCGTGACATCAAAGA** |
|  | **Reverse** | **ACAGGATTCCATACCCAAGAAG** |
| **h*PLGF*** | **Forward** | **GTTCAGCCCATCCTGTGTCT** |
|  | **Reverse** | **AACGTGCTGACAGAACGTCA** |
| **h*GAPDH*** | **Forward** | **GGAGCGAGATCCCTCCAAAAT** |
|  | **Reverse** | **GGCTGTTGTCATACTTCTCATGG** |
